# A preliminary open trial of olanzapine in paediatric acute and transient psychotic disorders

**DOI:** 10.4103/0019-5545.31618

**Published:** 2006

**Authors:** Vivek Agarwal, Prabhat Sitholey

**Affiliations:** * Assistant Professor, Department of Psychiatry, King George's Medical University, Lucknow 226003, Uttar Pradesh; ** Professor and Head, Department of Psychiatry, King George's Medical University, Lucknow 226003, Uttar Pradesh

**Keywords:** Olanzapine, paediatric, acute and transient psychotic disorders

## Abstract

**Background:**

Acute and transient psychotic disorders (ATPD) have been characterized by the development of florid psychotic symptoms within 2 weeks and complete remission of symptoms. Although there are no definite guidelines, these are usually treated by antipsychotic medication.

**Aim:**

This preliminary study examined the effectiveness of olanzapine in paediatric ATPD.

**Methods:**

In this 6-week open trial of olanzapine in paediatric ATPD, the patients were rated weekly on the Brief Psychiatric Rating Scale (BPRS), Clinical Global Impression (CGI) Scale and Dosage Record Treatment Emergent Symptom Scale (DOTES).

**Results:**

Twenty-three patients (11 males, 12 females; mean age 14.0±1.3 years; range 11–16 years) were included in the study. The mean olanzapine dosage was 12.7±3.9 mg/day (range 5–20 mg/day). All the patients showed significant improvement in 6 weeks. The results showed a significant decrease (p< 0.0001) in scores of BPRS (mean at baseline 46.2±7.0 to 21.4±3.9 at week 6). Severity of illness (CGI) decreased from 4.7±0.8 to 1.6±0.9 in 6 weeks. Also, global improvement (CGI) showed marked improvement in 14 (60.9%), good improvement in 8 (34.8%) and minimal improvement in 1 (4.3%) patient. Some common side-effects were dryness of mouth (*n*=14, 60.9%), increase in appetite (*n*=12, 52%), weight gain (*n*=12, 52%) and drowsiness (*n*=8, 34.8%). No patient developed extrapyramidal symptoms.

**Conclusion:**

Olanzapine was safe and effective in paediatric ATPD.

## INTRODUCTION

Acute and transient psychotic disorders (ATPD) have been characterized by the development of florid psychotic symptoms within 2 weeks and complete remission of symptoms.^1^ These may or may not be associated with stress. ATPD has been reported to occur more frequently in women, rural populations and developing countries.^2^,^3^ ATPD may persist for 1–3 months depending upon the specific diagnosis as per ICD-10.^4^ A study in 2000 reported 2–4 months' duration of ATPD in both developing and industrialized nations.^5^ Although ATPD is self-remitting, it has florid symptoms and it persists long enough to cause impairment to the patient and family. Therefore, ATPD should be treated. Although there are no definite guidelines, it is usually treated by antipsychotic medication. The use of typical antipsychotic drugs in children and adolescents (hereafter referred to as children unless specified) is limited because of the development of extrapyramidal symptoms (EPS), prolactin elevation and cognitive impairment. Atypical antipsychotics are used more often in children because of these drugs have a better tolerability profile.^6^

There are few studies on the use of antipsychotic medication in ATPD in children. An open trial of haloperidol^7^ and a placebo-controlled trial of risperidone^8^ are the only 2 studies known to the authors. In both the studies, all the children developed EPS and were given anticholinergic medication. Olanzapine, an atypical antipsychotic, has a similar receptor-binding profile as clozapine but produces less adverse effects. Olanzapine has been found safe and effective in the treatment of schizophrenia, bipolar disorder, pervasive developmental disorders and Tourette disorder in the paediatric population.^9^ Comparative studies of olanzapine with haloperidol and risperidone in adults have reported it to be better tolerated. Olanzapine produces significantly less EPS and patients require less anticholinergic medication than in the case of haloperidol or risperidone.^10^ The risk of new-onset tardive dykinesia was much less with olanzapine than with haloperidol or risperidone.^10^ To our knowledge, there is no study of olanzapine in ATPD in children. Therefore, this study aimed to examine the efficacy and tolerability of olanzapine in children and adolescents with acute psychosis.

## METHODS

### Sample

All children aged 6–16 years consecutively attending the child psychiatry department and presenting with symptoms suggestive of ATPD were assessed initially by a psychiatry postgraduate resident and then jointly with VA or PS. Children—both inpatients and outpatients—with a first episode of ATPD fulfilling the ICD-10^4^ criteria (F23) were included in the study. Children on antipsychotic treatment other than olanzapine were not included unless they showed no improvement in 3 weeks on adequate doses or had significant adverse effects. Medication of such children was cross-tapered off in 1week. Children with substance abuse and clinically significant physical illness were excluded. The study was approved by the institutional review board. Informed consent was obtained by parents of all children before their inclusion in the study.

### Assessment

VA assessed each patient again in detail, and a consensus diagnosis (VA and PS) was made using the ICD-10 criteria.^4^ Weight, blood pressure and pulse rate were recorded at baseline and thereafter weekly throughout the study period of 6 weeks. Routine blood (haemogram) and urine (biochemical and microscopic) investigations were done in all the cases to rule out any physical problem. The patients were assessed on the Brief Psychiatric Rating Scale^11^ (BPRS) and Clinical Global Impression^12^ (CGI) scale at baseline and thereafter weekly for 6 weeks. Adverse effects were recorded by using the Dosage Record Treatment Emergent Symptom Scale^13^ (DOTES) at baseline and weekly thereafter. VA provided the medication and did all the assessments weekly.

### Medication

The patients were started on olanzapine 5 mg/day in the first week. Thereafter, the dosage was increased or decreased every week by 2.5–5 mg up to a maximum of 20 mg/day depending upon the clinical improvement or adverse effects. Medications were administered to the patients daily directly under the supervision of one parent to ensure compliance. Concomitant lorazepam 2–4 mg was given during the day or at night for sedation, if needed. Trihexyphenidyl was given for EPS, where necessary.

### Statistical analysis

Statistical analysis was done using the paired *t*-test (two tailed) for comparison of baseline scores to scores at the end of weeks 1 to 6. Intent-to-treat analysis was done. The last observation of each patient was carried forward if the patient had at least 2 weeks of follow-up.

## RESULTS

### Sample characteristics and diagnosis

Twenty-three children (11 males, 12 females) with a mean age of 14.0±1.3 years (range 11–16 years) were included in the study. Twenty-two (5 inpatients and 17 outpatients) completed the trial while 1 was lost to follow-up after 3 weeks. Twenty patients were studying while 3 were illiterate. A family history of ATPD was present in the mothers of 2 patients and of mania in the sister of 1 patient. The mean duration of illness prior to inclusion in the study was 15.0±10.6 days. Eleven patients (47.8%) were diagnosed as having acute polymorphic psychotic disorder without symptoms of schizophrenia (F23.0), 5 (21.7%) with other ATPD (F 23.8), 3 (13.1%) with acute polymorphic psychotic disorder with symptoms of schizophrenia (F 23.1), 3 (13.1%) with acute schizophrenia-like psychotic disorder (F 23.2), and 1 (4.3%) with other acute predominantly delusional psychotic disorders (F 23.3).

### Medication dosage

Mean doses of olanzapine were 9.7±1.0, 12.1±2.9 and 12.7±3.9 mg/day at the end of week 2, 4 and 6, respectively. The dose in 1 patient was decreased from 15 mg/day to 12.5 mg/day after 4 weeks due to drowsiness. Concomitant lorazepam was needed in 11 patients (47.8%) a dosage of 2.5±0.9 mg/day in the first week. Nine children (39%) required lorazepam at a dosage of 2.2±1.1 mg/day in the second week. In the next 1–2 weeks, lorazepam was tapered off and it was given to 6 (26%) and 3 patients (13%) in week 3 and 4, respectively. Only 1 patient required trihexyphenidyl. This patient was previously prescribed haloperidol and she developed severe EPS without any improvement in her ATPD. Haloperidol was tapered off in 1 week and she was started on olanzapine. To manage EPS, trihexyphenidyl 6 mg/day was added in the first 2 weeks of the trial and then stopped.

### Response to treatment

All the patients showed improvement on olanzapine. The ratings of BPRS at the end of week 6 (Table 1) showed a significant improvement (t_22_ =18.15, p<0.0001) as compared to the baseline. Significant improvement was observed as early as the end of week 2 (t_22_ =7.63, p <0.0001). Severity of illness on the CGI scale also showed significant improvement (t_22_ =13.65, p<0.0001) at the end of week 6 as compared to baseline. A significant reduction in severity was seen at the end of week 2 (t_22_ =7.11, p<0.0001). The ratings of individual patients on the CGI scale showed marked improvement in 14 (60.9%), good improvement in 8 (34.8%) and minimal improvement in 1 (4.3%) patient (this patient was lost to follow-up after 3 weeks) at the end of 6 weeks.)

**Table 1:** BPRS and CGI scores at baseline and up to 6 weeks (*n*=23)

Scales	Baseline	End of week 1	End of week 2	End of week 3	End of week 4	End of week 5	End of week 6
**BPRS total score							
Mean (SD)	46.2 (7.0)	43.7 (7.7)	34.1 (5.8)*	29.1 (4.9)*	23.5 (4.6)*	22.5 (4.1)*	21.4 (3.9)*
***CGI severity of illness scores							
Mean (SD)	4.7 (0.8)	4.6 (0.9)	3.7 (0.9)*	2.9 (0.8)*	2.3 (1.1)*	1.7 (0.9)*	1.6 (0.9)*
CGI global improvement							
Mean (SD)	NA	3.7 (0.5)	2.8 (0.8)	2.1 (0.7)	1.6 (0.7)	1.4 (0.6)	1.4 (0.6)

NA: Not applicable

* p<0.0001

** BPRS: Brief Psychiatric Rating Scale (18 items)

*** CGI: Clinical Global Impression scale

### Adverse effects

All the children tolerated olanzapine well. Adverse effects were seen in 19 patients (82.6%). Some common adverse effects were dryness of mouth (*n*=14, 60.9%), increase in appetite (*n*=12, 52%), weight gain (*n*=12, 52%) and drowsiness (*n*=8, 34.8%). Other adverse effects were headache (*n*=6, 26%), dizziness (*n*=2, 8.6%) constipation (*n*=2, 8.6%), blurring of vision (*n*=1, 4.3%), and decreased appetite (*n*=1, 4.3%). No patient developed EPS. The adverse effects were usually mild. Only 2 patients (8.6%) developed moderate drowsiness and increased appetite. A significant increase (t_22_ =3.81, p<0.001) in weight was observed at the end of the study (mean at baseline 32.7±4.6 kg to 34.0±5.3 kg at the end of the study) in 8 male and 4 female patients. Weight gain was 1 kg in 4 patients, 2 kg in 2 patients, 3 kg in 4 and 5 kg in 2 patients. It was gradual over 6 weeks (average weight gain 6.6%). Clinical assessment did not warrant repeat haematological laboratory investigations during or at the end of the study in any patient.

## DISCUSSION

This study shows that olanzapine is effective in the treatment of ATPD in children. Recent studies on olanzapine in schizophrenic children and adolescents without resistance to drug treatment have shown that olanzapine is beneficial.^14^–^16^ A prospective open trial of olanzapine in 12–17-year-old adolescents with schizophrenia reported significant reduction (p<0.0005) in PANSS total scores and CGI-severity of illness at the end of 8 weeks. The most common adverse effects reported were increased appetite, weight gain and sedation. EPS was observed in 2 patients.^16^ These results are comparable with those of our study in which the BPRS total scores and CGI-severity of illness showed a significant reduction (p<0.0001) at the end of 6 weeks. Some common side-effects seen in our study were dryness of mouth, sedation, increased appetite and weight gain. No patient in our study developed EPS.

The remission rate (95.7%) in our study is comparable with or even better than the remission rates in acute psychosis treated with haloperidol^7^ (*n*=27, 10–20 mg/day; 88.9% response) and with risperidone^8^ (*n*=12, 4–6 mg/day; 75% response). Also, olanzapine was well tolerated by all the adolescents in this study. No patient developed EPS and concomitant lorazepam was needed in only 47.8% of patients at a dose of 1–4 mg/day for the initial 1–2 weeks. One may argue that the improvement in these children (47.8%) occurred because of lorazepam. On comparing the improvement beyond week 2 (mean BPRS score 35.6±5.8) in these children (*n*=11), there was a significant improvement at week 4 (mean BPRS score 25.3±5.3, p<0.001) and at week 6 (mean BPRS score 23.0±4.6, p <0.0001) as compared to week 2. So, it can be confidently said that the improvement in these children was because of olanzapine as they improved significantly beyond week 2.

In the studies on haloperidol^7^ and risperidone^8^ all the patients developed EPS and had to be given trihexyphenidyl. Eleven per cent of patients dropped out of the risperidone study because of EPS. Weight gain was also observed in 55% of patients in the risperidone study. In our study, weight gain was observed in 52% of patients. Weight gain with olanzapine has also been reported in treatment studies in schizophrenic children.^17^ Weight gain should not be of much concern in cases of ATPD as long-term maintenance treatment is not required. This weight gain can be controlled by dietary measures during and after treatment and after recovery by physical activity. The dosage of olanzapine in our study (12.7±3.9 mg/day) was also comparable to the dosage of Findling *et al*.^16^ (12.4±5.3 mg/day) and to the usual recommended dose of 10 mg/day for children and adolescents.^18^ Therefore, olanzapine may be used in the treatment of paediatric ATPD.

## LIMITATION

Limitations of this study are like those of any open and uncontrolled trial. However, the use of placebo in children with ATPD with florid symptoms poses an ethical dilemma. In the risperidone study, 42.8% patients dropped out in the placebo group as they became unmanageable despite being given lorazepam.^8^ This study did not monitor the plasma glucose levels. There are a few reports of hyperglycaemia with olanzapine in adolescents.^19^ However, treatment emergent hyperglycaemia has been described with typical and atypical antipsychotics and olanzapine is no exception.^20^ Also, hyperglycaemia is reversible without any intervention after stoppage of olanzapine. However, caution must be taken while prescribing olanzapine for children with diabetes or those with a family history of diabetes.

## CONCLUSION

This preliminary study shows that olanzapine may be a good first-line antipsychotic drug in paediatric ATPD because of its effectiveness and tolerability. It has a better side-effect profile and patients require less concomitant medication. Also, it has a convenient dosing schedule.

This suggests that further randomized, controlled trials on larger samples are required to confirm the efficacy and safety of olanzapine in paediatric ATPDs.
